# Detection of Glial Fibrillary Acidic Protein in Patient Plasma Using On-Chip Graphene Field-Effect Biosensors, in Comparison with ELISA and Single-Molecule Array

**DOI:** 10.1021/acssensors.1c02232

**Published:** 2021-12-15

**Authors:** Lizhou Xu, Sami Ramadan, Oluwatomi E. Akingbade, Yuanzhou Zhang, Sarah Alodan, Neil Graham, Karl A. Zimmerman, Elias Torres, Amanda Heslegrave, Peter K. Petrov, Henrik Zetterberg, David J. Sharp, Norbert Klein, Bing Li

**Affiliations:** † Department of Materials, Imperial College London, London SW7 2AZ, U.K.; ‡ Department of Brain Sciences, Imperial College London, London W12 0BZ, U.K.; § Care Research & Technology Centre, UK Dementia Research Institute, London W12 0BZ, U.K.; ∥ Graphenea Semiconductor, Paseo Mikeletegi 83, San Sebastián 20009, Spain; ⊥ UK Dementia Research Institute at UCL, University College London, London WC1E 6BT, U.K.; # Department of Neurodegenerative Disease, UCL Institute of Neurology, London WC1E 6BT, U.K.; ∇ Department of Psychiatry and Neurochemistry, Institute of Neuroscience and Physiology, the Sahlgrenska Academy at the University of Gothenburg, Mölndal 43141, Sweden; ○ Clinical Neurochemistry Laboratory, Sahlgrenska University Hospital, Mölndal 43141, Sweden; ◆ Hong Kong Centre for Neurodegenerative Diseases, Hong Kong 999077, China

**Keywords:** single-molecule array, graphene field-effect transistor, biosensor, glial fibrillary acidic protein, traumatic brain injury blood biomarker

## Abstract

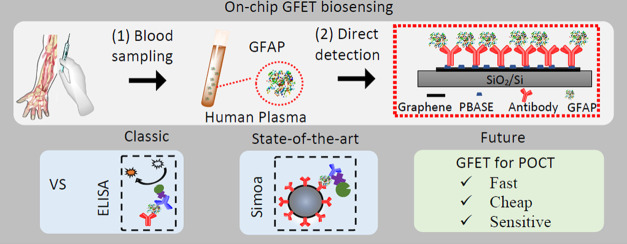

Glial fibrillary acidic protein (GFAP) is a discriminative blood biomarker for many neurological diseases, such as traumatic brain injury. Detection of GFAP in buffer solutions using biosensors has been demonstrated, but accurate quantification of GFAP in patient samples has not been reported, yet in urgent need. Herein, we demonstrate a robust on-chip graphene field-effect transistor (GFET) biosensing method for sensitive and ultrafast detection of GFAP in patient plasma. Patients with moderate–severe traumatic brain injuries, defined by the Mayo classification, are recruited to provide plasma samples. The binding of target GFAP with the specific antibodies that are conjugated on a monolayer GFET device triggers the shift of its Dirac point, and this signal change is correlated with the GFAP concentration in the patient plasma. The limit of detection (LOD) values of 20 fg/mL (400 aM) in buffer solution and 231 fg/mL (4 fM) in patient plasma have been achieved using this approach. In parallel, for the first time, we compare our results to the state-of-the-art single-molecule array (Simoa) technology and the classic enzyme-linked immunosorbent assay (ELISA) for reference. The GFET biosensor shows competitive LOD to Simoa (1.18 pg/mL) and faster sample-to-result time (<15 min), and also it is cheaper and more user-friendly. In comparison to ELISA, GFET offers advantages of total detection time, detection sensitivity, and simplicity. This GFET biosensing platform holds high promise for the point-of-care diagnosis and monitoring of traumatic brain injury in GP surgeries and patient homes.

Traumatic brain injury (TBI) affects millions of people every year, and it has become one of the major burdens for the healthcare systems in many developed countries.^1^ Symptom assessment and clinical examination currently form an important part of TBI assessment; however, factors such as disease complexity, differences in practitioner expertise, incorrect referrals, and long waiting times^2,3^ cause significantly delayed diagnosis. In light of this, an accurate and reliable blood biomarker-based point-of-care (POC) diagnosis is extremely promising because: (1) blood biomarkers could quickly reflect the dynamic progress of TBI after the trauma-risk activity.^4^ This happens before the symptoms appear and is critical for early-stage diagnosis. (2) The blood samples can be accessed through the widely accessible acquisition and handling infrastructures,^5^ through less invasive and less expensive procedures, such as venous blood collection in GP surgeries and finger-prick blood collection in patients’ homes. This allows the at-risk individual to be screened and referred to the most suitable specialist at their earliest possible stage.

Great progress has been made in the discovery of TBI blood biomarkers, as reported by Shahim et al.^4^ Among them, glial fibrillary acidic protein (GFAP) is an astrocytic cytoskeletal protein that appears post-injury in blood. The fluctuation of GFAP concentration accurately reflects the progress of TBI, which makes it an ideal biomarker for the TBI early diagnosis and monitoring. It is also a biomarker for a range of other neurological diseases,^6−10^ including glioblastoma multiforme, multiple sclerosis, intracerebral hemorrhage, and Alzheimer’s disease.^11^ Classic methods for the detection of GFAP include enzyme-linked immunosorbent assay (ELISA)^12^ and Western blot techniques.^13^ However, their sensitivities and detection ranges are inadequate to cover the clinically relevant concentration from a few femtomolars up to the nanomolar level.^9,14^ While more advanced assays have been developed and available in some clinical laboratories, i.e., mass spectrometry^15^ and single molecular array (Simoa) technology,^11^ these techniques require complicated fluorescent labeling processes, demanding laser excitation, and signal capture systems, as well as highly skilled operational personnel and high maintenance cost. These reasons limited the accessibility of the above methods to serve in GP surgeries and patients’ homes.

Recently, a growing number of biosensing approaches have been published for the detection of GFAP, with an aim to achieve point-of-care (POC) detection. For example, electrochemical sensors based on anti-GFAP antibodies^16^ or molecularly imprinted polymer technology^17^ have been proposed for GFAP detection in buffer solution with the limit of detection (LOD) values of 10^1^ fM and 10^2^ pM. An organic field-effect transistor with a LOD of 10^1^ pM has been reported for GFAP detection in buffer solution.^18^ However, there are a limited number of papers reporting a biosensor device for GFAP detection in samples with matrix effects, such as serum or plasma. One example is a fluorescence sensor using anti-GFAP antibodies functionalized with carbon dots for labeling and signal reporting, reaching a LOD of the order of pM in 25 pg/mL in buffer solution.^19^ This method was then applied to the determination of GFAP in four spiked human serum samples at 0.1–0.4 ng/mL range, and the results showed good recovery. A very recent study showed a ultrahigh-frequency surface acoustic wave (SAW) sensor for the successful GFAP detection in fetal bovine serum (FBS) matrix at a concentration as low as 35 pM.^20^ A polyethylenimine-modified graphene oxide electrochemical immunosensor was reported for the detection of GFAP in the dynamic range of 1 pg/mL to 100 ng/mL in spiked serum within 45 min.^21^ These are the only few examples of GFAP detection in a nonbuffer environment using biosensors. However, they all tested GFAP under the spiked serum/FBS condition, rather than in patient samples (with naturally produced GFAP). For clinical diagnosis and POC screening, more sensitive biosensing methods and detection data toward demonstrations in patient samples are required to measure GFAP biomarker GP surgeries and patients’ home.^6^

Over the last decade, graphene field-effect transistor (GFET) has emerged as a promising detection method for the early diagnosis of disease and point-of-care testing (POCT)^22,23^ for several reasons: graphene exhibits an extremely high surface-to-volume ratio and tunable electronic properties that make it sensitive to any charged molecules near its surface;^24^ graphene offers fast response due to its high mobility. In addition, graphene is biologically compatible and can be directly functionalized without the need for new functionalization steps or damaging its sp^2^ network.^25^ Furthermore, GFET biosensors consume very low amounts of power and have great potential for mass production. GFET biosensors have been utilized for ultralow detection of a variety of biological species including proteins, exosomes, DNAs, viruses, and other disease biomarkers.^26−32^ However, so far, there is no report of the detection of GFAP in patient samples for neurological diseases using the GFET technology. Here, we recruited six patients with moderate–severe TBI (defined by the Mayo classification), and we demonstrated an on-chip GFET biosensing platform for ultrasensitive and ultrafast detection of GFAP with the LOD down to 20 fg/mL (400 aM) in clean phosphate-buffered saline (PBS) and 231 fg/mL (4 fM) in patient plasma. The sensing performance has been compared for the precise detection of GFAP in patient plasma samples using the state-of-the-art Simoa technology, classic ELISA, and our on-chip graphene FET, which represents the trend of future biosensing technology.

## Materials and Methods

### Patient Samples

Patients with moderate–severe TBI, defined by the Mayo classification,^33^ were recruited from a trauma center in London as part of the BIO-AX-TBI study (Health Research Authority approval reference 17/LO/2066).^34^ Inclusion criteria were age between 18 and 80 at time of injury and a diagnosis of moderate–severe TBI. Exclusion criteria were preexisting neurological disease, previous TBI requiring hospitalization, significant drug or alcohol abuse, or pregnancy. Venous blood was sampled peripherally or by central access line if available, using ethylenediaminetetraacetic acid (EDTA)-coated tubes for plasma samples. After 30 min at room temperature, samples were centrifuged at 2500× *g* at 4 °C, transferred into 1.4 mL aliquots, and frozen at −80 °C. The healthy control plasma samples (PS0) were provided by Merck (U.K.) with a negligible GFAP concentration.

### Simoa

Plasma GFAP concentration was measured at University College London using the Simoa platform (HD-x instrument) and the Simoa GFAP Discovery Kit according to the manufacturer’s instructions (Quanterix, Billerica, MA). First, samples were added neat to the plate and then diluted × 4 on board the instrument. The samples were then incubated with the mixture of capture antibody-modified magnetic microbeads and biotinylated conjugate for 35 min. The microbeads were incubated with streptavidin-ß-galactosidase (SBG) for 5 min, followed by a washup step, and then resuspended in a resorufin ß-d-galactopyranoside (RGP) substrate solution to generate optical signal. The concentrations of GFAP were obtained using a four-parameter 1/Y2 weighted curve fit with seven calibrator points between 1.37 and 1000 pg/mL. These calibrator points were measured from a serial dilution of concentrated calibrator in the assay kit. Three plasma samples were used as the quality controls with GFAP concentrations of 283.0 pg/mL (high), 61.0 pg/mL (medium), and 13.6 pg/mL (low). Coefficient of variation (CV) was calculated as a ratio of the standard deviation (SD) to the mean of the duplicate Simoa measurements, as a measure of the accuracy of the measured GFAP concentration. CV values below 30% were considered acceptable.

### Biofunctionalization of On-Chip GFET

The biofunctionalization mainly includes an incubation step of a linker molecule 1-pyrenebutanoic acid succinimidyl ester (PBASE), which has pyrene groups that binds to graphene through π–π interaction, and *N*-hydroxysuccinimide (NHS) ester, an amine-reactive reagent, that extends from the graphene surface to react with primary amines present on the GFAP antibody. First, the GFET devices were incubated with PBASE (10 mM in dimethylformamide (DMF) (Sigma-Aldrich)) for 2 h at room temperature and then gently rinsed in DMF to remove excessive PBASE from the graphene surface and dried in N_2_. Then, the unlabeled capture antibody from Human GFAP Matched Antibody Pair Kit (ab222279, Abcam, U.K.) was used. Multiple GFAP antibodies have been tested, and the chosen one showed the highest sensitivity to blood GFAP. The antibody incubation solution was prepared using 1× PBS at pH 8.4 to a concentration of 0.25 mg/mL. Droplets of 20 μL of the incubation solution were added onto the chip surface and left overnight in a humidified environment at 4 °C. The chip was then sequentially rinsed in 1 × PBS and deionized (DI) water and dried with N_2_. The chip was blocked using 3% bovine serum albumin (BSA) in 1 × PBS for 1 h, then rinsed with PBS and DI water, and dried with N_2_ prior to electrical measurement.

### Electrical Detection of GFAP Using GFET

Immediately following the incubation and cleaning of GFET sensor chip (model S20), the electrical measurements were performed in 0.001 × PBS solution (d1000 PBS to ensure the low ionic strength) using a Keysight B1500 semiconductor analyzer. Source–drain voltage was fixed at 20 mV, and electrolyte gate was swept from 0.2 to 0.9 V at a sweeping rate of 30 mV/s, rendering source–drain currents in the order of 10^1^ μA and a power consumption of ≈ 0.2 μW. The working concentrations of GFAPs were prepared by a 10-time serial dilution from the stock solution in d1000 PBS. For the detection in patient plasma samples, the samples were first diluted 100 or 1000 times in d1000 PBS and then measured using GFET sensors as mentioned above.

### Overview of the Detection Principles

**Figure 1:**
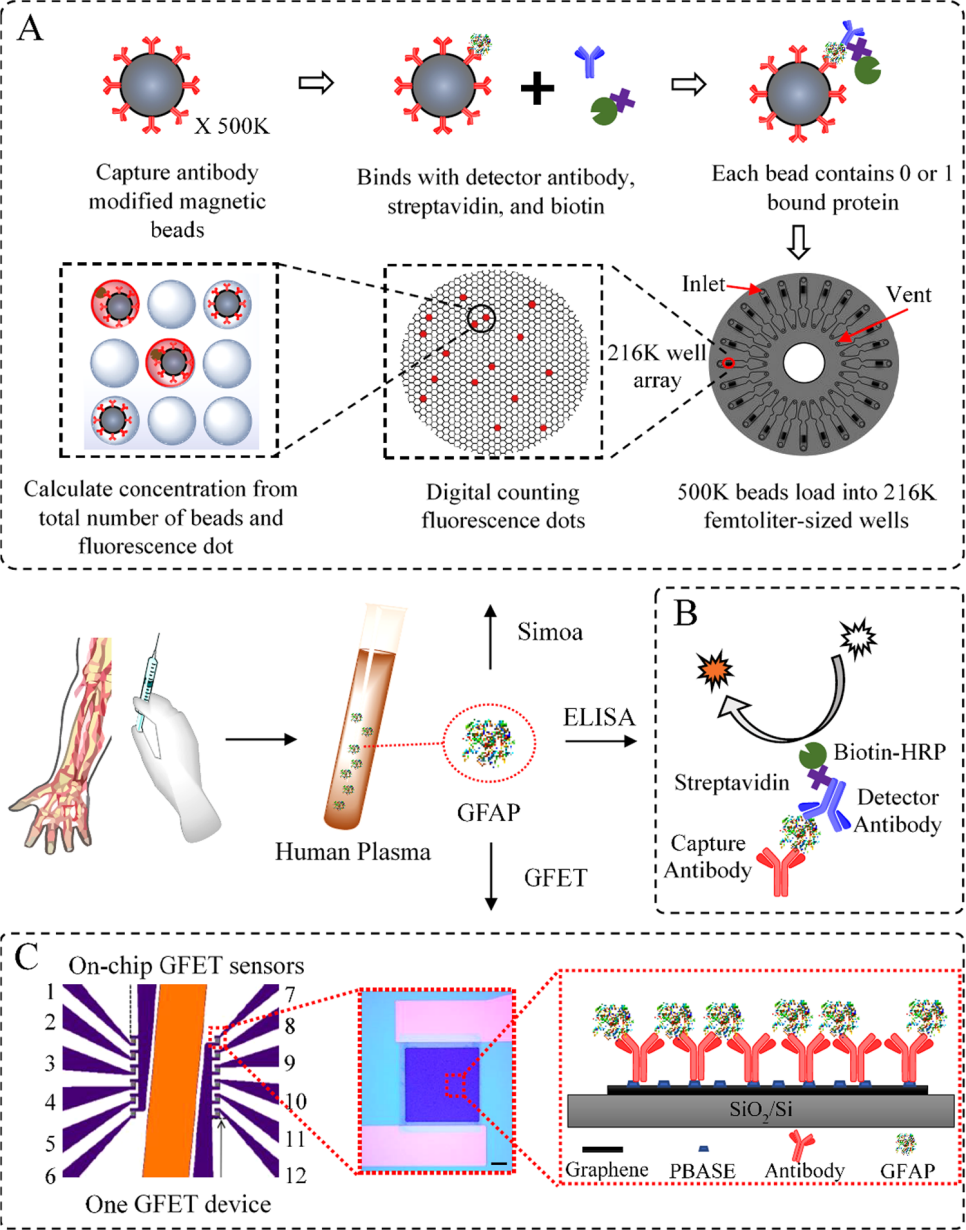
Schematic of the methods for GFAP detection. (A) State-of-the-art Simoa technology relies on the effective binding between 500 K antibody-modified magnetic beads and the GFAP molecules at a low concentration. The GFAP concentration is determined by digital counting of the fluorescent signal from 216 K femtoliter-sized wells (for sample with high concentrations, there is also analogous signal quantification). (B) Classic sandwich ELISA uses an HRP-based colorimetric detection. The concentration is determined by the integration of TMB color changes. (C) On-chip GFET biosensing platform uses anti-GFAP functionalized graphene channel as a sensing element. The nonencapsulated reference electrode (orange) allows liquid gating without external electrode. Detection is based on the shift of Dirac point in response to the extent of antigen binding, which is linked to the GFAP concentration within a solution.

The working mechanisms of the three methods that we used for direct GFAP detection in patient plasma samples are presented in Figure 1. Figure 1A shows the workflow of the Simoa technology, which is believed to be the gold standard diagnostic method in laboratorial settings. Briefly, the patient samples are first mixed with over 500 K anti-GFAP modified magnetic beads to ensure high-efficiency binding between the anti-GFAP-modified beads and the GFAP molecules. The detection antibodies and the fluorescence-generating agents are then coupled with the immobilized GFAP molecules. At low concentrations, each bead contains one bound protein, or none. After the beads are loaded into an array of femtoliter-sized wells (216 K microwells, each large enough to hold one bead), the protein concentration is determined by digitally counting the beads, where the fluorescent signal is proportional to the total number of beads on the array. The high binding efficiency and the way to count signal presence or absence (rather than integrating) offer a LOD at the single-molecule level.

Figure 1B shows the schematic of sandwich ELISA, which is an immunological assay developed in the 1970s, and is most commonly used to measure antibodies, antigens, proteins, etc. in biological samples. ELISA is used as a classic method for comparison in this study, and the experimental details are presented in Section S1 in the Supporting Information (SI).

To achieve POC detection, methods with simple procedure, user-friendly equipment, and fast sample-to-result time would be preferable. Therefore, the on-chip GFET biosensor is proposed here for direct, sensitive, and reliable detection of GFAP in patient samples within minutes (Figure 1C). The GFET sensor array consists of two areas, each with six GFET devices. Each chip includes a nonencapsulated gold reference electrode (on-chip) to allow the low-power liquid gating. This eliminates the requirement of the external gate electrode and further reduces the gate power consumption, making the GFET platform more aligned with POC technologies. The graphene surface of each GFET is modified with the PBASE and the GFAP antibodies, which are used for capturing the target GFAP proteins in a liquid environment.^26,27^ The binding of GFAP with antibody triggers the positive shift of Dirac point due to the negatively charged GFAP molecules at the neutral pH.^35^ The electrical response of each device can be determined using liquid gates, i.e., the source–drain current is measured as a function of the liquid gate voltage applied.

## Results and Discussion

### Detection of GFAP Using Simoa Technology

The Simoa detection results of GFAP in patient plasma samples are summarized in Table 1. For the detection of each sample, two tests have been conducted in parallel, with the GFAP concentration ranging from 36 to 56 424 pg/mL. Except for the sample PS1, which shows a CV value of 11.0%, all other tests have presented CV values below 4.6%, showing low variation between repeat readings of the same sample. The LOD of Simoa assay for all GFAP detection has been determined to be 1.18 pg/mL, according to the internal calibration running within the HD-X analyzer. This demonstrates the excellent detection performance of the Simoa method for patient sample analysis.

**Table 1:** Detection of GFAP in Human Plasma Using Simoa

		GFAP concentration (pg/mL)					95% CI (pg/mL)	
sample group	sample no	test 1	test 2	mean	CV%	*P* value	upper limit	lower limit
healthy control	PS0	0	0	0	0	N/A	0	0
TBI patient	PS1	39	33	36	11.0	0.0529	0	74
TBI patient	PS2	1822	1792	1807	1.2	0.0053	1616	1997
TBI patient	PS3	4206	4484	4345	4.5	0.0204	2578	6111
TBI patient	PS4	9978	10 437	10 108	4.6	0.0143	7291	13 123
TBI patient	PS5	26 516	22 673	23 094	2.6	0.0496	179	49 009
TBI patient	PS6	55 883	56 965	56 424	1.4	0.0061	49 549	63 298

### Detection of GFAP Using ELISA

**Figure 2:**
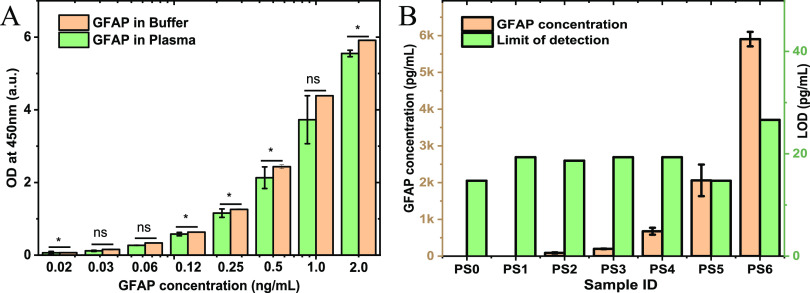
Validation of anti-GFAP and the detection of GFAP in seven patient plasma samples using ELISA (six patient samples and one healthy control). (A) Comparison between mean ± standard deviation (SD) of optical densities (OD) at 450 nm absorbance in assay buffer solution (pale orange) and control plasma solution (pale green) when spiked with known concentrations between 0.02 and 2.0 ng/mL of GFAP. All concentrations were measured in duplicate. ns = *p* > 0.05; * = 0.01 < *p* < 0.05. (B) Concentrations of GFAP detected in the healthy control sample (PS0) and six patient samples (PS1–PS6) measured using ELISA (left axis), and the assay LODs (right axis). All samples were measured in duplicate.

The most important factors in biosensing platforms are the affinity and the selectivity of antibodies, which determine the function and reliability of one assay. In this project, multiple commercial anti-GFAPs have been sourced and tested. The one which shows the highest affinity and selectivity has been used in ELISA and the GFET development. Figure 2A presents the validation results of this anti-GFAP as a capture antibody for the detection of GFAP in plasma. The GFAP standards for ELISA with concentrations from 20 pg/mL to 2 ng/mL have been prepared with serial dilutions in both the assay buffer and in the control plasma. Compared with the GFAP detections in buffer solution, no significant difference in optical densities (OD) values can be observed for the GFAP detection in plasma at concentrations of 0.03, 0.06, and 1 ng/mL, while the detections at the other five concentrations showed differences up to 12% in plasma samples, as presented in Figure 2A. This indicates the good affinity and selectivity of the anti-GFAP.

This GFAP antibody was then used for GFAP detection in seven plasma samples, including one healthy control (PS0) and six patient samples (PS1–PS6). The GFAP concentrations for the healthy control and one of patient samples (PS1) are below the LOD of ELISA assays (14–26 pg/mL). And the other patient plasma samples (PS2, PS3, PS4, PS5, and PS6) have shown GFAP concentrations of between 88 and 5904 pg/mL, as shown in Figure 2B. Repeat readings of samples with detectable GFAP concentrations showed low variation (CV values below 30%), statistically different readings from the control sample (*p* < 0.01, as determined by one sample *t*-test), and 95% confidence intervals within the detection limits of our ELISA platform. The detailed ELISA results are presented in Table S1, together with their fittings in Figure S1.

### Functionalization and Characterization of On-Chip GFET Platform

**Figure 3:**
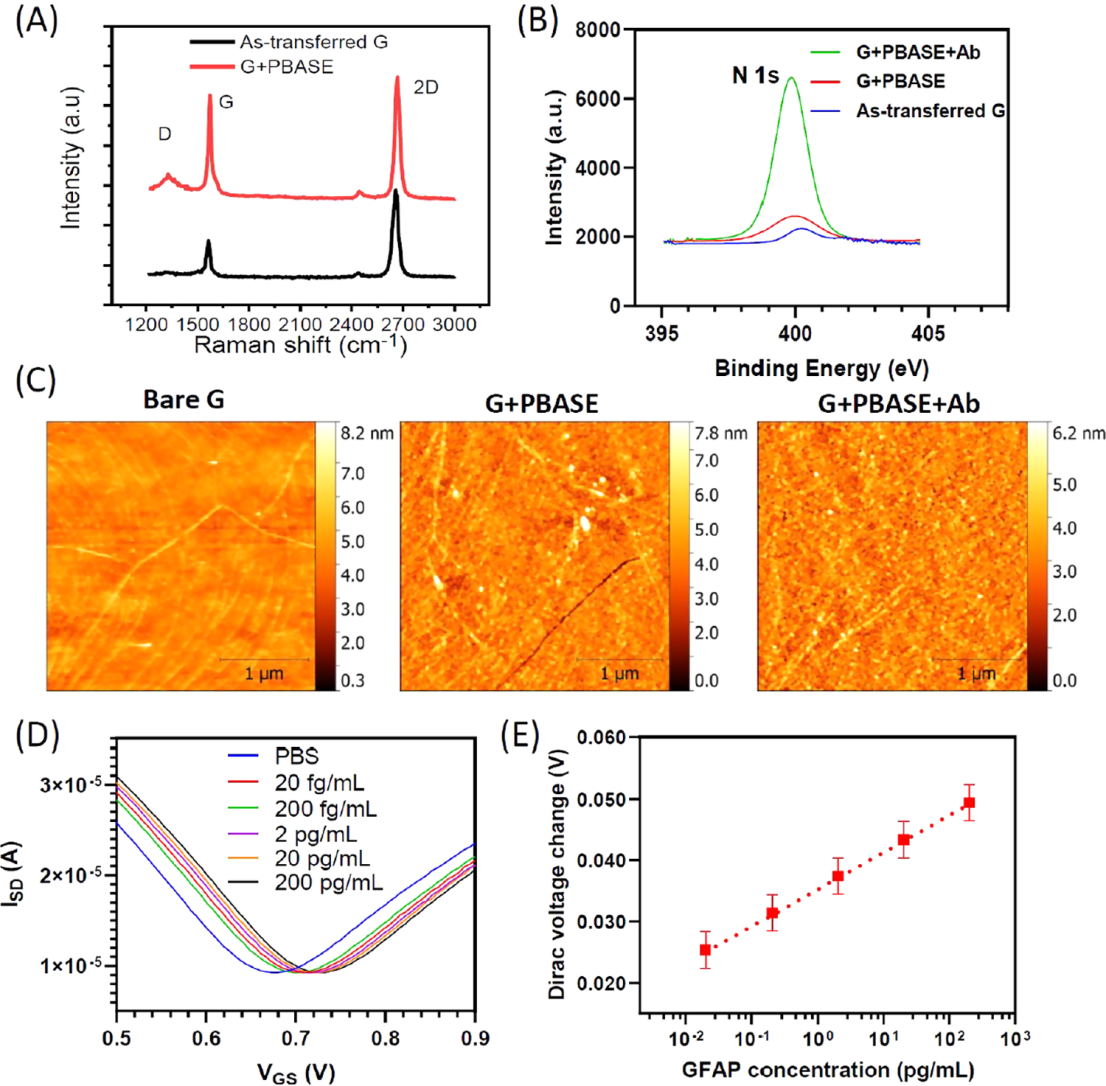
Characterization of the GFET biosensor. (A) Raman spectra confirming the existence of PBASE as the linker molecule. (B) X-ray photoelectron spectroscopy (XPS) of N 1s peak confirming the biofunctionalization of PBASE and antibody on the graphene surface. (C) Atomic force microscopy (AFM) images for the characterization of PBASE and antibody on the graphene surface. (D) Transfer curves of GFET in PBS with and without GFAP biomarkers. (E) Responses of GFET sensors for the detection of GFAP biomarker in PBS buffer.

Several characterization techniques were performed to confirm the successful functionalization of PBASE and anti-GFAP on the graphene sensor surface. The influence of PBASE on graphene properties was investigated using Raman spectroscopy. Figure 3A shows a comparison of Raman spectra taken on the as-transferred graphene and the graphene after PBASE functionalization. The Raman spectrum of as-transferred graphene features 2D and G peaks at 2687 cm^–1^ and at 1588 cm^–1^, indicating the high quality of monolayer graphene. In addition, the small defect peak at around 1341 cm^–1^ indicates a high quality of graphene after transfer. After PBASE functionalization, the G band exhibits another shoulder peak at 1616 cm^–1^ assigned to the pyrene groups in PBASE binding to the graphene.^26,36^ Furthermore, it was observed that a significant increase in D peak occurs after PBASE modification (*I*_D_/*I*_G_ < 0.1 for as-transferred graphene and around 0.3 after PBASE), which is attributed to localized vibrational modes of the PBASE interacting with extended phonon modes of graphene.^37^

X-ray photoelectron spectroscopy (XPS) has been used to further confirm the functionalisation of PBASE and anti-GFAP on the graphene surface. Figures 3B and S3 show the evolution of N 1s and C 1s spectra after each functionalisation step, respectively. The high-resolution N 1s spectrum show a significant increase in the N 1s peak at 400 eV after anti-GFAP conjugation. Furthermore, the C 1s spectrum is broadened after anti-GFAP functionalisation and becomes more asymmetric. It also shows higher intensity peaks at C–C at 284.8 eV, C–O/C–N at 286 eV, and O–C=O at 288 eV due to the large number of amine and amide groups present on the antibodies. Atomic force microscopic was used to validate the presence of PBASE on the graphene surface. Figure 3C shows an increase in surface roughness from 0.45 nm for as-transferred graphene to 0.8 nm after anti-GFAP modification of graphene surface.

After confirmation of the attachment of PBASE and the antibody on the graphene surface, the GFET biosensor was then used for the detection of GFAP proteins in PBS in concentrations from 20 fg/mL to 200 pg/mL. Figure 3D shows the transfer curves of GFET in PBS with and without GFAP biomarkers. The GFAP samples show a significant right shift of Dirac point in comparison to the PBS control, and the larger concentration resulted in a larger shift. The positive shift in Dirac voltage after GFAP binding is consistent with the negative charge effect of GFAP, which causes p-doping in graphene channel. The Dirac voltage change of each sample versus the GFAP biomarker concentration in PBS for the GFET sensor in the detection of GFAP was plotted in Figure 3E. A linear relation (*y* = 0.0057*x* + 0.0188, *R*^2^ = 0.9966) could be found, with the limit of detection of 20 fg/mL (400 aM). The Sips model in eq S2 is used to fit the shift in Dirac voltage as a function of GFAP concentration in Figure 3E. The fitting parameter values *A* = 0.1 V, and average *K*_D_ = 1.880 ng/mL (37.6 pM). The value of *K*_D_ is consistent with the value of dissociation constant for antibody-protein interaction.^38^

### Detection of GFAP in Patient Plasma Using the On-Chip GFET Biosensor

**Figure 4:**
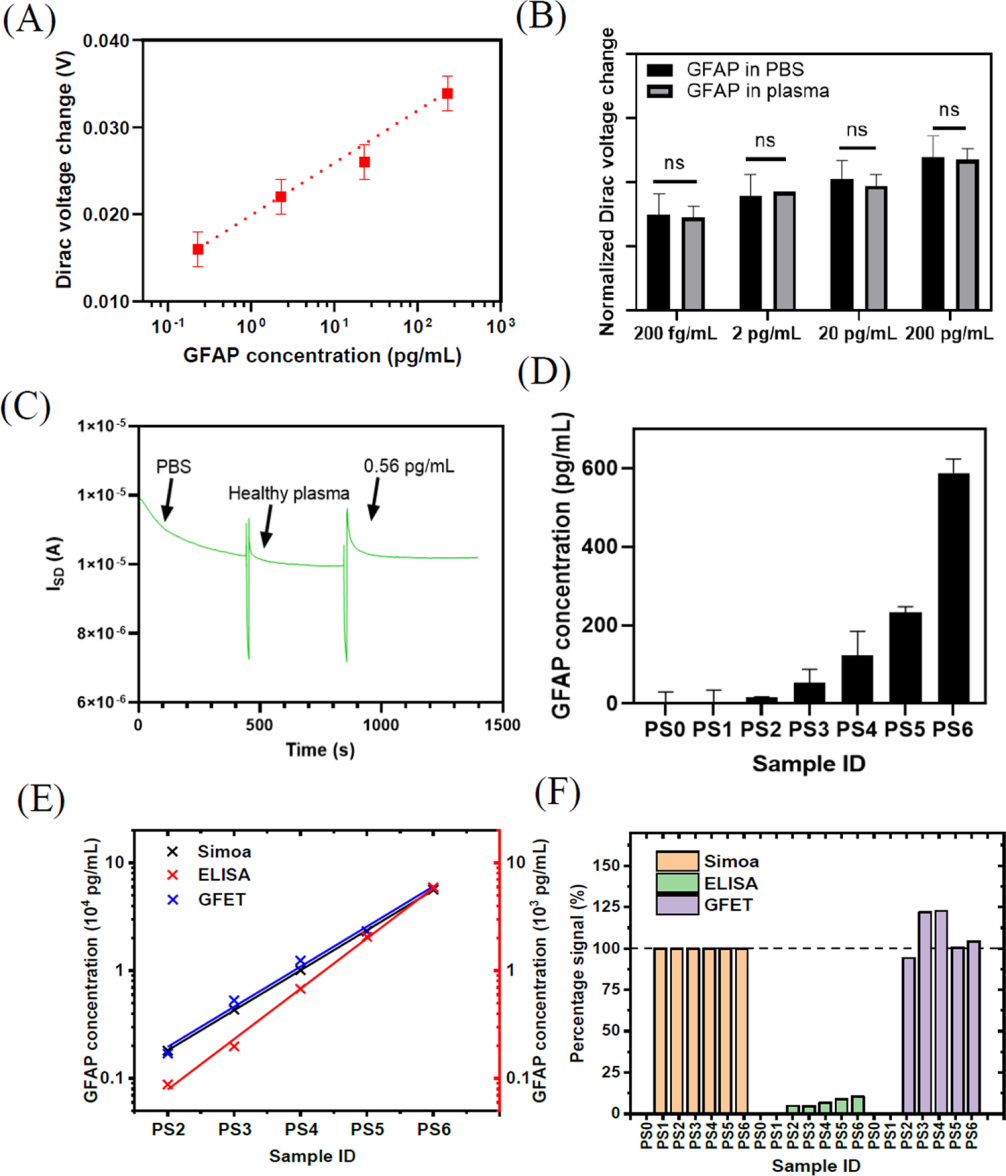
Detection of GFAP in patient plasma using the GFET biosensor. (A) Calibration curve of the GFET biosensor for the GFAP detection in plasma. *n* = 3. (B) Signal intensity comparison between the tests in PBS and in the plasma for the same concentration order of magnitude illustrating excellent selectivity of the GFAP biosensor. (C) Real-time response of the GFAP biosensor for the detection of GFAP in plasma. Significant change seen in the curve for the sample of 0.56 pg/mL in comparison to the healthy control, suggesting that the sensor is able to respond to the sub-pg/mL (4 fM) level of GFAP in plasma. (D) Measurement results of six patient samples and one control plasma sample by the GFET. (E) Correlation of GFAP concentration measured by Simoa, ELISA, and GFET. GFAP concentration measured by GFET results showed significant correlation with those measured by Simoa and ELISA (*p* < 0.0001 and *p* < 0.001, respectively). The PS1 data are not fitted, as it is only available for Simoa. (F) Signal percentage of GFAP concentration measured by GFET and ELISA in comparison to Simoa as a reference. The GFAP concentrations in both PS0 and PS1 measured by ELISA and GFET are below their LODs.

We next directly measured the GFAP concentration in patient plasma samples using this GFET biosensor. Figure 4A shows the calibration curve of the GFET biosensors for the GFAP detection in plasma (*y* = 0.0056*x* + 0.0110, *R*^2^ = 0.9563). The GFET sensor has a good linear response in the range from 2.3 × 10^2^ fg/mL to 2.3 × 10^2^ pg/mL, with a LOD (defined as the lowest concentration that was tested) of 2.3 × 10^2^ fg/mL (4 fM). The up-detection range is limited by the high viscosity of plasma. We further compared the signal intensity between the GFAP detection in the PBS and in the plasma samples for the same concentration order of magnitude, as shown in Figure 4B. There was no significant difference between the Dirac voltage change (normalized) detected in plasma samples and those in PBS. Since all plasma samples contain many homological biomarkers, for example, S100B at 10^1^-10^2^ pg/mL and neurofilament light at 10^0^–10^2^ pg/mL (both measured by Simoa). The detection results indicate that the sensor has little interference from other non-target biomarkers in the plasma, illustrating excellent selectivity of the GFAP biosensor. This is in great agreement with the ELISA results, which show good affinity and selectivity of the anti-GFAP. The sensor also shows good real-time response in the addition of either PBS samples or patient samples. As shown in Figure 4C, the addition of a plasma sample containing 0.56 pg/mL of GFAP caused a significant increase in the current intensity between the source and drain electrodes compared to the healthy control, which has no GFAP. In addition, the stable current signal can be obtained around 200 s (∼3 min) post injection of the sample, which proves the rapid detection for patient samples using the GFET biosensor. This again demonstrates that the GFET sensor has analytical resolution down to the order of 10^–1^ pg/mL level in patient plasma samples.

Furthermore, we tested six patient samples and one control healthy plasma sample (three repeats for each sample) using the GFET method. Each patient sample was diluted in d1000 PBS 100 times (to reduce the viscosity) to obtain a testing sample with an appropriate GFAP concentration. The measured results are shown in Figure 4D. The control sample showed a negative signal, and five patient samples showed increasing signal with increasing concentration of GFAP, according to the corresponding GFAP values from Simoa data. However, the sensor showed a signal below the LOD for the sample PS1, indicating very low GFAP concentration. As far as we know, this GFET biosensor is the first technology showing ultrahigh sensitivity down to the 2.3 × 10^2^ fg/mL (4 fM) level for the detection of GFAP in patient plasma samples, which is much lower than the cutoff value for typical clinical assessment.^6,20,39^

The GFAP concentration in patient plasma samples measured by state-of-the-art Simoa, classic ELISA, and GFET platforms are shown in Figure 4E. The GFAP concentrations measured by different methods follow the same trend, i.e., PS2 has the lowest detectable GFAP concentration and PS6 has the highest detected GFAP concentration. Quantitatively, compared with Simoa, the GFET method shows a high correlation coefficient of 0.9991 for the five patient samples (PS2–PS6), but nondetectable GFAP in PS1. Compared with ELISA, the GFET method presents a correlation coefficient of 0.9968. These high correlation coefficients prove that the GFET biosensor is accurate and reliable for the detection of GFAP in patient plasma. In addition, the GFAP concentration measured by Simoa were normalized to be 100% for each plasma sample (normalized data shown in Table S2). Compared with Simoa results, ELISA results showed a signal percentage between 4.5 and 10.5%, while GFET results presented a signal percentage between 94.1 and 122.7% for the corresponding samples, as shown in Figure 4F. This also indicates that GFAP concentrations measured by GFET has better agreement with those measured by Simoa. Simoa is considered a gold standard technology, which proves that the GFET biosensor is a better detection tool for GFAP in patient plasma samples than the ELISA method.

### Comparison of GFAP Detection Using Different Methods

The classic ELISA technique is supported by widely available kits from industry, which makes it a convenient method for biomarker detection in patient samples. Nevertheless, a relatively long detection time (of at least a few hours), limited sensitivity, and the requirement of a laboratory environment precludes its use in GP surgeries or patients’ home. The supersensitive state-of-the-art Simoa technology has the potential to improve the accuracy of the GFAP detection process; however, high costs, the demanding detection instrument, and specialized procedures are the main limitations for the use of Simoa as a POC solution. The limitations of both ELISA and Simoa in biomarker detection could be countered by GFET-based biosensor platforms, which are able to provide high sensitivity, specificity, short sample-to-result time, relatively low costs, and user-friendliness. With these advantages, the GFET biosensor proposed in this work has great promise to be further developed as an accurate and standardized blood testing solution for the detection of GFAP, and possibly other biomarkers, in primary care settings to combat neurological diseases.

Table 2 summarizes other recent biosensor work reported for GFAP detection in nonbuffer samples, including, e.g., spike serum, plasma, etc. Our GFET biosensor achieved a LOD of 2.3 × 10^–1^ pg/mL for direct GFAP detection in patient plasma. This is more sensitive than the carbon dot-based fluorescence sensor (LOD of 25 pg/mL in buffer),^19^ the graphene-PEI electrochemical sensor (LOD of 1 pg/mL in artificial serum),^21^ and the ultrahigh-frequency surface acoustic wave (SAW)-based sensor (LOD of 35 pM or 1.75 ng/mL in spiked GFAP in FBS).^20^ The total detection time (sample-to-result) of our GFET sensor is less than 15 min including incubation, which is faster than the three sensors mentioned above, with more than 15 min for the SAW sensor, around 45 min for the electrochemical sensor, and about 3 h for the fluorescence sensor, respectively. To the best of our knowledge, these three are the only demonstrations of GFAP detection in a nonbuffer environment. Hence, our work is the first biosensing example of direct GFAP clinical detection. In comparison to other reported sensors, this on-chip, real-time, and label-free GFET biosensing platform, demonstrated in patient plasma samples, shows obvious advantages in detection time and sensitivity.

**Table 2:** Comparison of Reported Biosensors for GFAP Detection in Nonbuffer Samples^a^

detection method	sample	recognition element	LOD (pg/mL)	detection time	potential in POCT	ref.
Simoa	plasma	Ab	1.18	1–3 h	/	this work
ELISA	plasma	Ab	15	5–8 h	+	this work
GFET	plasma	Ab	0.23	<15 min	+++	this work
SAW^1^	spiked serum	Ab	35 pM	>15 min	+	(20)
CLAISA^2^	spiked serum	Ab	25 (in buffer)	∼3 h	+	(19)
electrochemical	spiked serum	Ab	1	45 min	++	(21)

a Note: 1. surface acoustic wave. 2. CD-linked antibody immunosorbent assay.

## Conclusions

In this work, we have successfully demonstrated an on-chip GFET platform as a real-time, label-free, and selective tool for the detection of neurological diseases biomarker GFAP with ultrahigh sensitivity. Our on-chip GFET sensors were able to directly detect GFAP in patient plasma samples with the LOD down to the femtomolar level without signal amplification within minutes. The outstanding performance shown by this on-chip GFET biosensor compared with conventional or state-of-the art detection technologies such as ELISA or Simoa in terms of simplicity, real-time, and ultralow LOD holds great promise for point-of-care and advanced brain diseases diagnosis.

## Supporting Information Available

Sandwich ELISA detection of GFAP, Raman characterization of the functionalization process, XPS characterization of the functionalization process, Sips fitting of Dirac voltage shift as a function of GFAP concentration, and comparison of the detection results by Simoa, ELISA, and GFET (PDF)
