# Supplementary material for: Detection of Glial Fibrillary Acidic Protein in Patient Plasma Using On-Chip Graphene Field-Effect Biosensors, in Comparison with ELISA and Single-Molecule Array
Source: ACS Sens. 2021 Dec 15;7(1):253–62. doi: 10.1021/acssensors.1c02232 (PMC8805154; doi:10.1021/acssensors.1c02232)
Supplement: Supplementary file 1 — se1c02232_si_001.pdf [file se1c02232_si_001.pdf]

Supplementary Information

Detection of Glial Fibrillary Acidic Protein in Patient Plasma using On-chip Graphene Field-effect Biosensors, in Comparison with ELISA and Single Molecule Array

*Lizhou Xu[1, #], Sami Ramadan[1, #], Oluwatomi E. Akingbade[2, 3], Yuanzhou Zhang[1], Sarah Alodan[1], Neil Graham[2, 3], Karl A. Zimmerman[2, 3], Elias Torres[4], Amanda Heslegrave[5, 6], Peter K. Petrov[1], Henrik Zetterberg[5, 6, 7, 8, 9], David J. Sharp[2, 3], Norbert Klein[1, *], Bing Li[2, 3, *]*

[1] Department of Materials, Imperial College London, London, SW7 2AZ, UK

[2] Department of Brain Sciences, Imperial College London, W12 0BZ, UK

[3] Care Research & Technology Centre, UK Dementia Research Institute, W12 0BZ, UK

[4] Graphenea Semiconductor, Paseo Mikeletegi 83, San Sebastián, 20009, Spain

[5] UK Dementia Research Institute at UCL, University College London, London, WC1E 6BT, UK

[6] Department of Neurodegenerative Disease, UCL Institute of Neurology, London, WC1E 6BT, UK

[7] Department of Psychiatry and Neurochemistry, Institute of Neuroscience and Physiology, the Sahlgrenska Academy at the University of Gothenburg, Mölndal, 43141, Sweden

[8] Clinical Neurochemistry Laboratory, Sahlgrenska University Hospital, Mölndal, 43141, Sweden

[9] Hong Kong Centre for Neurodegenerative Diseases, Hong Kong, 999077, China

# These authors contributed equally.

* Corresponding authors:

Prof. Dr. Norbert Klein, n.klein@imperial.ac.uk

Dr. Bing Li, b.li@imperial.ac.uk

Sandwich ELISA Detection of GFAP

Sandwich ELISA was performed using the Human GFAP Matched Antibody Pair Kit (Abcam, Cambridge UK) containing capture antibody (rabbit monoclonal anti-GFAP antibody), biotinylated detector antibody (rabbit monoclonal anti-GFAP antibody) and purified human GFAP protein as the standard. Blocking buffer, streptavidin conjugated horse radish peroxidase solution (HRP), 3,3',5,5'-tetramethylbenzidine (TMB) solution and stop solution (containing sulfuric acid) were purchased from Abcam, Cambridge (UK). Reagents were diluted to working concentration in blocking buffer containing 1% BSA and 0.05% Tween20. PBS-Tween 20 (0.05%) solution was used as a wash buffer in this procedure. All incubations occurred at room temperature (RT) (20-25°C) on the Heidolph™ Titramax 1000 orbital plate shaker (400 rpm) (Fisher Scientific, Leicestershire, UK).

Briefly, all reagents were bought to RT before use. The Nunc MaxiSorp™ flat-bottom 96-well plates (Life Science Technologies, MA, USA) were coated with anti-GFAP capture antibody and incubated for 120 min. After incubation, the wells were washed with blocking buffer for 120 min to prevent non-specific binding. Wells were washed with the wash buffer before the addition of standard samples or clinical plasma samples. Purified human GFAP standard

protein was used to validate antibody-antigen binding. Purified human GFAP protein was diluted to 2000 pg/mL and then serially diluted (two-fold) in blocking buffer to create a standard curve. Plasma samples were thawed at RT for 30 min and then serially diluted (two-fold) in blocking buffer. If required (based on concentration determined by Simoa), plasma samples were diluted to 2000 pg/mL (the upper detection limit of the assay) prior to serial dilution. Blocking buffer was used as a blank control for standards and plasma samples. Standard protein dilutions, plasma samples and blocking buffer were added to wells in duplicate and plates were incubated for 120 min. After incubation, plates were washed with wash buffer and then incubated with biotinylated anti-GFAP detector antibody for 60 min. Wells were washed with wash buffer before the addition of a streptavidin conjugated HRP solution for 60 min. The plates were washed for the final time and then plates were incubated with TMB solution in the dark for 20 min to allow for quantification of the immobilised (detector) antibody-complexes. Finally, stop solution was added to wells and incubated for 1 min. The absorbance of each sample and standard was immediately read at 450 nm wavelength using a Varioskan Lux Multimode microplate reader (Thermo Fisher Scientific, Dartford, UK) with SkanIt Software for Microplate Readers, Table S1. GFAP standard protein and plasma samples were tested in duplicate.

Table S1. Detection of GFAP in seven clinical human plasma samples (one healthy control, PS0, and six patients, PS1 - PS6) using ELISA

Sample No	GFAP concentration (pg/mL)			CV%	P value	95% CI (pg/mL)		LOD (pg/mL)
	Test 1	Test 2	Mean			Upper limit	Lower limit	
PS0	Below LOD	Below LOD	N/A	N/A	N/A	N/A	N/A	14.7
PS1	Below LOD	Below LOD	N/A	N/A	N/A	N/A	N/A	19.3
PS2	94	83	88	15.9	0.0002	69	108	18.7
PS3	217	180	198	3.9	0.0007	175	222	19.3
PS4	746	611	678	13.8	<0.0001	585	772	19.3
PS5	21745	1949	2061	20.9	0.0037	1272	2851	14.7
PS6	6790	5019	5905	3.3	<0.0001	5544	6265	26.6

Note: Mean GFAP detection and resulting coefficient of variation (CV%), significant

difference from control sample (p value, calculated from one sample t-test), 95% confidence interval (lower limit and upper limit) (95% CI), and the limit of detection (LOD), pg/mL, of all seven samples measured by ELISA. N/A refers to instances in which calculations are not applicable as not calculated due to GFAP concentration being below the readable limit of the assay. All samples were measured in duplicate.

The mean +/- standard deviation (SD) average density of duplicate readings of serial dilutions of the GFAP standard protein were fitted using a four-parameter logistic curve:

$$y = y^0 + \left(\frac{A}{w\sqrt{\pi/2}}\right)\left(exp^{-2\left(\frac{x - xc}{w}\right)^2}\right) \quad S1$$

Where y^0 is the baseline offset value, *xc* is the centre of the peak, *w* is equal to 2 times the standard deviation of the Gaussian distribution of the curve and *A* is the area under the curve. Unknown GFAP concentrations from clinical samples were extrapolated from the four-parameter logistic curve derived from the GFAP protein standard (Fig. S1) and the averages calculated to give the final GFAP concentration of PS2-PS6.

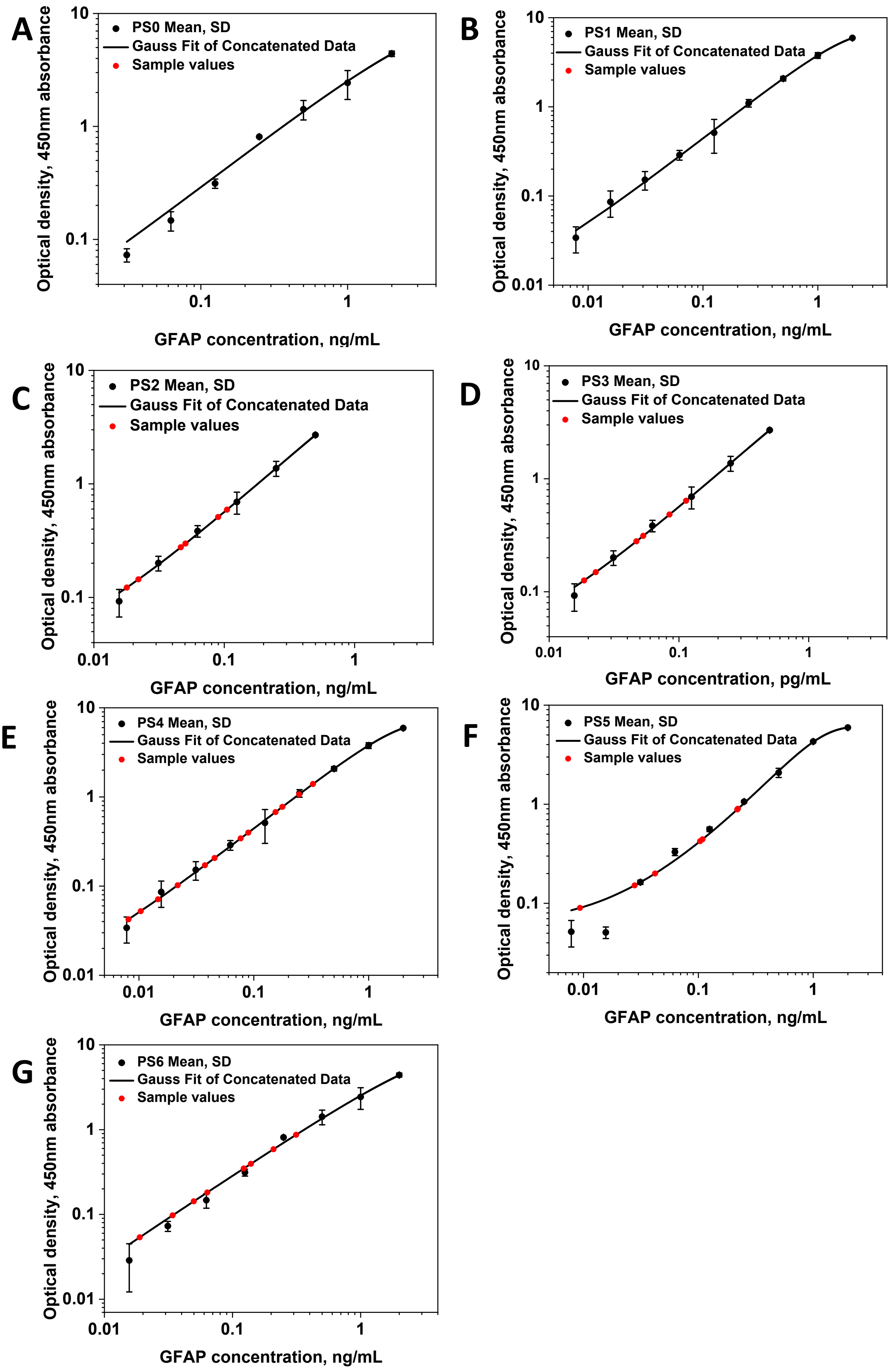
A
PS0 Mean, SD
Gauss Fit of Concatenated Data
Sample values
Optical density, 450nm absorbance
GFAP concentration, ng/mL
B
PS1 Mean, SD
Gauss Fit of Concatenated Data
Sample values
Optical density, 450nm absorbance
GFAP concentration, ng/mL
C
PS2 Mean, SD
Gauss Fit of Concatenated Data
Sample values
Optical density, 450nm absorbance
GFAP concentration, ng/mL
D
PS3 Mean, SD
Gauss Fit of Concatenated Data
Sample values
Optical density, 450nm absorbance
GFAP concentration, pg/mL
E
PS4 Mean, SD
Gauss Fit of Concatenated Data
Sample values
Optical density, 450nm absorbance
GFAP concentration, ng/mL
F
PS5 Mean, SD
Gauss Fit of Concatenated Data
Sample values
Optical density, 450nm absorbance
GFAP concentration, ng/mL
G
PS6 Mean, SD
Gauss Fit of Concatenated Data
Sample values
Optical density, 450nm absorbance
GFAP concentration, ng/mL

Figure S1. Detection of GFAP in six clinical plasma samples and one healthy control using ELISA method. The optical density of serial dilutions of samples PS0, N=0 (A); PS1, N=0 (B); PS2, N=5 (C); PS3, N=3 (D); PS4, N=7 (E); PS5, N=4 (F); PS6, N=4 (G) was compared to the optical density of the standard (GFAP in buffer). Standard curves were established using known concentrations of GFAP in buffer fitted with a four-parameter logistic curve. GFAP in samples PS0 and PS1 were below the detectable limit of the assay. All concentrations were measured in duplicate.

Raman characterisation of functionalisation process

Raman spectroscopy measurements were performed using a Witec spectrometer with laser wavelength of 532 nm (excitation energy $E_L = \hbar w_L = 2.33$ eV) through an optical fiber, and an objective lens of 100×, NA = 0.8, and laser spot of 0.4 μm. The laser power was kept below 2 mW and spectral resolution was ~3 cm^{-1}; the Raman peak position was calibrated based on the Si peak position at 520.7 cm^{-1}. The D, G, and 2D peaks were fitted with Lorentzian functions. Fig. S2. Shows I_D/I_G ratio before and after functionalisation of graphene with PBASE. An increase of the I_D/I_G is observed after graphene modification with PBASE which can confirm the presence of PBASE (see manuscript section 3.3).

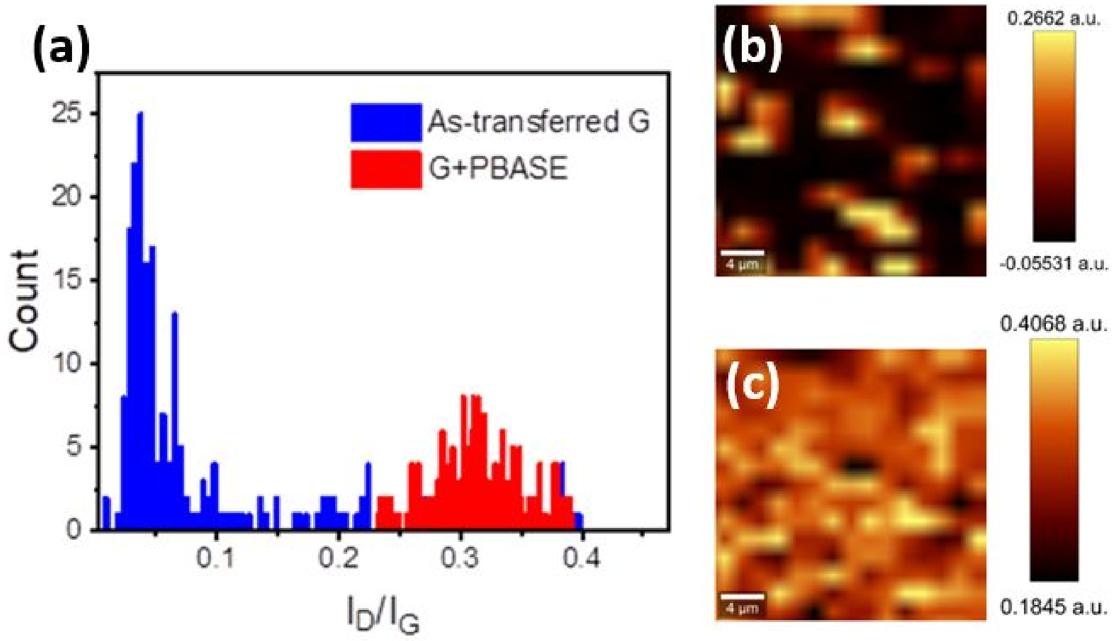

Figure S2. Comparison of Raman I_D/I_G intensity ratio between as-transferred graphene and graphene functionalised with 10 mM PBASE. (a) Histogram shows the I_D/I_G ratio for as-transferred graphene and graphene after functionalisation with PBASE. The statistics shows an increase in I_D/I_G after modification of graphene with PBASE. (b) Raman map of I_D/I_G for as-transferred graphene. (c) Raman map of I_D/I_G for PBASE modified graphene.

XPS characterisation of functionalisation process

XPS experiments and measurements were performed with K-Alpha+ and an Al radiation source (hv = 1486.6 eV) in an ultrahigh vacuum chamber for spectroscopic analysis with a base pressure of 5×10^{-8} mbar. XPS is used to confirm the presence of PBASE and Ab on graphene surface (See manuscript section 3.3). High resolution C1 s spectra show higher intensity peaks at C–C at 284.8 eV, C–O/C–N at 286, and O–C═O at 288 eV after incubation of Ab due to the large number of amine and amide groups present on the antibodies.

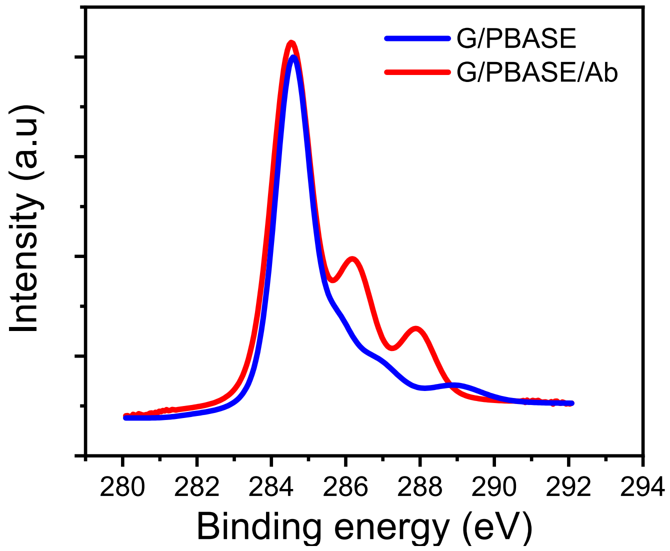

Figure S3. C 1s XPS spectra of graphene with PBASE, and with PBASE + GFAP antibody.

Fitting the shift in Dirac voltage as a function of GFAP concentration

Sips model was used to fit the GFET response and is given by:

$$\Delta V_{DV} = V_{Max} \frac{(\frac{C}{K_D})^a}{1 + (\frac{C}{K_D})^a} \qquad S2$$

Where V_{Max} is the maximum in Dirac voltage with all antibody sites are occupied, c is the GFAP concentration, a is the characteristics of distribution function, and K_D is the dissociation constant. The fitting parameter values V_{Max}=0.1 V, and average K_D= 1.88 ng/mL.

Comparison of the detection results by Simoa, ELISA and GFET

Table S2. Comparison of the detection results for GFAP in patient samples by three methods: Simoa, ELISA and GFET

**Sample**	**Conc. by Simoa**		**Conc. By ELISA**		**Conc. By GFET**
	Measured results	Samples for GFET (diluted 100 times)	Measured results	Normalised concentration (dilute 100 times)	Measured results
**PS0**	0	0	0	0	0
**PS1**	36	0.36	0	0	0
**PS2**	1807	18	88	0.88	17
**PS3**	4345	43	198	1.98	53
**PS4**	10108	101	678	6.78	124
**PS5**	23094	230	2061	20.61	232
**PS6**	56424	564	5904	59.04	588
